# Anti-osteoporosis medications in persons with and without Parkinson disease in Finland

**DOI:** 10.1007/s11657-026-01780-z

**Published:** 2026-10-05

**Authors:** Miriam T. Y. Leung, Marja-Leena Lamidi, Miia Tiihonen, Sirpa Hartikainen, J. Simon Bell, Justin P. Turner, Anna-Maija Tolppanen

**Affiliations:** 1 Centre for Medicine Use and Safety, Faculty of Pharmacy and Pharmaceutical Sciences, Monash University, Melbourne, Australia; 2 School of Pharmacy, University of Eastern Finland, Kuopio, Finland; 3 Kuopio Research Center of Geriatric Care, University of Eastern Finland, Kuopio, Finland; 4 Faculty of Health Sciences, University of Eastern Finland, Kuopio, Finland; 5 Faculty of Pharmacy, Laval University, Quebec, Canada

**Keywords:** Hip fracture, Parkinson’s disease, Antiresorptive medication, Osteoporosis

## Abstract

***Summary*:**

Contrary to reports on under-treatment in persons with Parkinson’s disease, our study found similar anti-osteoporosis medication usage before and after their hip fractures among Finns with and without Parkinson’s disease. Additional preventive measures may be needed to mitigate the high risk of hip fractures among persons with Parkinson’s disease.

**Purpose:**

Parkinson’s disease (PD) is associated with a doubled risk of hip fracture. However, previous reports suggest under-recognition of osteoporosis among persons with PD. Our study aimed to assess the annual prevalence of anti-osteoporosis medications among persons with and without PD in relation to diagnosis of PD and hip fractures.

**Methods:**

Our nationwide matched cohort design included 33,153 persons diagnosed with incident PD between 2000 and 2009 in Finland and 1:2 matched comparison cohort. Persons with previous hip fractures were excluded. Annual prevalences of anti-osteoporosis medications were calculated for each year between 5 years before and 10 years after diagnosis of PD, and between 5 years before and 5 years after the incident hip fractures.

**Results:**

Prevalence of anti-osteoporosis medications was higher in persons with PD (3.62%, 95% confidence interval [CI] 3.28–3.98%) than those without PD (2.91%, 95% CI 2.70–3.14%), starting from 3 years before the diagnosis. Among individuals with incident hip fractures (1475 [13%] persons with PD and 1371 [6%] persons without PD), the annual prevalence were similar among persons with PD and without PD, peaking within 1 year after hip fractures (with PD: 20.3%, 95% CI 18.0–22.8%; without PD: 21.0%, 95% CI 18.5–23.6%).

**Conclusion:**

Contrary to reports on under-treatment in persons with PD, our study found similar anti-osteoporosis medication usage before and after their hip fractures among Finns with and without PD. Additional preventive measures, such as regular review of fall risk, is needed to mitigate the doubled risk of hip fracture in PD.

## Introduction

Neurological disorders are the leading cause of disability globally [1]. Among all neurological disorders, Parkinson’s disease (PD) had the fastest growing prevalence, disability and deaths [2]. PD has become the second most common neurodegenerative disease, following Alzheimer’s disease [2].

It is well-established that persons with PD had a higher risk of hip fractures. In addition to the higher risk of falls from poorer gait and balance control [3, 4], the higher risk of hip fracture has been attributed to the higher risk of osteoporosis [5, 6]. Persons with PD had consistently been shown to have lower bone mineral density than persons without PD [5]. The poorer bone health may be due to impaired mobility leading to reduced physical activity, low body weight, or bone loss as a part of the pathophysiology [7]. However, previous studies have found that the current risk prediction algorithm underestimated the risk of hip fracture in persons with PD [8, 9], suggesting that the risk of hip fracture was under-recognized in persons with PD.

Our study aimed to investigate the annual prevalence of anti-osteoporosis medication use in persons with and without PD in Finland from 5 years before to 10 years after the diagnosis of PD. The secondary objective was to investigate the annual prevalence of anti-osteoporosis medication use among persons with and without PD who had an incident hip fracture, from 5 years before to 5 years after incident hip fracture.

## Methods

### Study population and data sources

Our study was conducted in the nationwide matched cohort of persons with and without Parkinson’s disease in Finland (FINPARK) [10]. This dataset contained all persons registered in the Special Reimbursement Register entitled for the reimbursement of anti-Parkinson drugs, and age- and diagnosis-based exclusion criteria were applied to improve the sensitivity of diagnosis as described previously [10, 11]. This resulted in including 22,189 persons with PD. On the diagnosis date (index date), each person with PD were matched by age ± 1 and sex to two comparison persons without PD at index date (Fig. S1). During the study period, the diagnosis criteria in Finland was consistent with that of United Kingdom Parkinson’s Disease Society Brain Bank criteria [12].

The dataset consisted of records from Care Register for Healthcare, Prescription Register and Statistics Finland, which were linked using the personal identification number unique to each resident in Finland. Care Register for Healthcare contains diagnosis made within hospital admission and specialized healthcare outpatient visits. Diagnoses were coded according to the International Classification of Diseases and Related Health Problems, 8th (ICD-8), 9th (ICD-9) and 10th Revision (ICD-10), during the years of 1972–1986, 1987–1995, and 1996–2019 respectively. Prescription Register contains dispensing records of reimbursed medications in community pharmacy. Statistics Finland has information on all deaths registered in Finland.

The study plan for FINPARK study was approved by the national health and social data permit authority FINDATA. Under the Act on secondary use of health and social data, informed consent or additional ethical approvals were not required as the study participants were not contacted, and all data were pseudonymized before the research team received access to data.

To allow 10-year follow-up after index date for each study participant, we restricted our cohort to persons diagnosed with PD between 2000 and 2009. To ensure we were investigating the primary prevention of hip fracture, the study was further restricted to incident hip fractures. We excluded persons who experienced a hip fracture (ICD-8 [820], ICD-9 [820] and ICD-10 [S72.0-S72.2]) prior to the start of follow-up at 5 years before index date.

### Prevalence of anti-osteoporosis medications

To assess the use of anti-osteoporosis medications, we retrieved dispensing records for anti-osteoporosis medications (World Health Organization Anatomical Therapeutic Classification [ATC] M05B, H05BA), including bisphosphonate (ATC M05BA, M05BB), denosumab (ATC M05BX04), or calcitonin (ATC H05BA).

Annual prevalence was defined as the number of persons with at least one dispensing of the medication, divided by the number of persons with ≥ 60 days in the community in that year. The restriction was to allow sufficient time for dispensing the medications from community pharmacy. The number of days in the community was defined as the number of days from start of year till censoring, subtracted by the numbers of days admitted to hospital. Follow-up was censored at death, end of year, end of study, and developing PD for comparison cohort. To ensure 1:2 matching, we also excluded matched counterparts of persons excluded from the base population of that year.

For our primary objective to assess the annual prevalence of anti-osteoporosis medications relative to progression of PD, annual prevalences were computed from 5 years before to 10 years after diagnosis of PD.

For our secondary objective to assess anti-osteoporosis medication for primary and secondary prevention of hip fracture, annual prevalences were computed from 5 years before to 5 years after hip fractures among persons with incident hip fractures. No further matching was conducted to avoid bias from overmatching and to retain representativeness of our target population.

### Statistical analysis

Annual prevalence of anti-osteoporosis medication use was calculated as a percentage. The confidence interval (CI) was estimated using Clopper-Pearson exact method. Comorbidities at baseline were identified using records from Special Reimbursement Register since 1972 to index date, Care Register for Healthcare and Prescription Register within 5 years before index date [11] (Table S1). Median and interquartile range (IQR) were reported for age. Differences across persons with and without PD and persons with and without hip fractures were assessed using Pearson’s Chi-squared test and Wilcoxon rank sum test. To understand the variation across sex, subgroup analysis stratified by sex were conducted. Sensitivity analysis was also conducted with additional censoring at hip fracture for primary objective. All analyses were performed using R (version 4.0.0).

## Results

Our final cohort consisted of 11,051 persons with PD and 22,102 matched comparison persons without PD. Median age of the cohort was 72 (IQR 64–78) years old and were composed of 54% (*n* = 18,000) male. Compared to matched comparison persons at 5 years before diagnosis, persons with Parkinsons’ disease were more likely to be diagnosed with cardiovascular diseases, stroke, osteoporosis and depression, but were less likely to be diagnosed with rheumatoid arthritis, asthma/chronic obstructive pulmonary disease, and had similar records of diabetes, renal failure, previous fractures and substance abuse (Table S2). The prevalences of comorbidities were in line with our previous studies in this cohort [13, 14]. Bisphosphonates were the most commonly used anti-osteoporosis medications. Low use of calcitonin was found in our study due to its low efficacy and it has been out of market in Finland since 2012 [15].

**Fig. 1:**
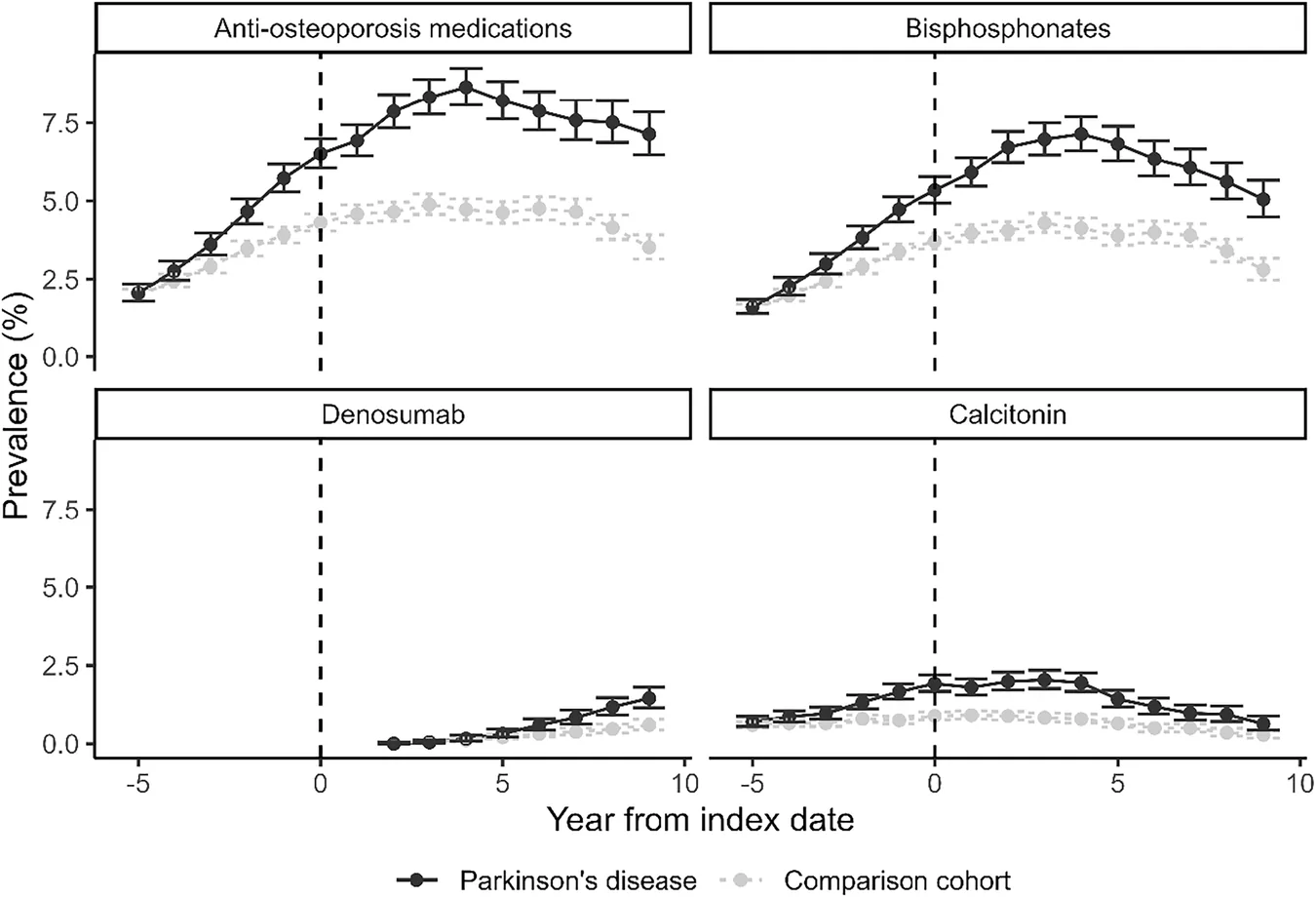
Annual prevalence of dispensing in persons with and without Parkinson’s disease from 5 years before to 10 years after diagnosis of Parkinson’s disease

The prevalences for all anti-osteoporosis medications were higher among persons with PD. The divergence in prevalences for anti-osteoporosis medications started to emerge 3 years before index date (PD: 3.62% 95% CI 3.28–3.98%; without PD: 2.91% 95% CI 3.24–3.73%), and peaked at 4 after index date (PD: 8.67%, 95% CI 8.09–9.27%; without PD: 4.73%, 95% CI 4.40–5.07%) and remained elevated till end of follow-up (PD: 7.14%, 95% CI 6.47–7.85%; without PD, 3.52%, 95% CI 3.15–3.93%) (Fig. 1). The prevalence and divergence in prevalence of denosumab steadily increased till the end of follow-up, once it became observable in our cohort (Fig. 1).

Females with or without PD had higher annual prevalences of anti-osteoporosis medications than males with or without PD throughout the follow-up period (Fig. S2). Females with PD had higher prevalence of anti-osteoporosis medications than males with PD at the date of PD diagnosis (females with PD: 11.7%, 95% CI 10.1–12.7%; males with PD: 2.11%, 95% CI 1.76–2.51%). While both sexes had similar trends before index date, prevalence of anti-osteoporosis medications for females with PD peaked at 4 years after index date (15.5%, 95% CI 14.4–16.6%), contrasting that of males with PD, which remained similarly elevated till the end of follow-up, peaking at 8 years after PD diagnosis (3.16%, 95% CI 2.58–3.82%) (Fig. S2). Sensitivity analysis with additional censoring upon occurrence of hip fracture showed similar trends (Fig. S3).

**Fig. 2:**
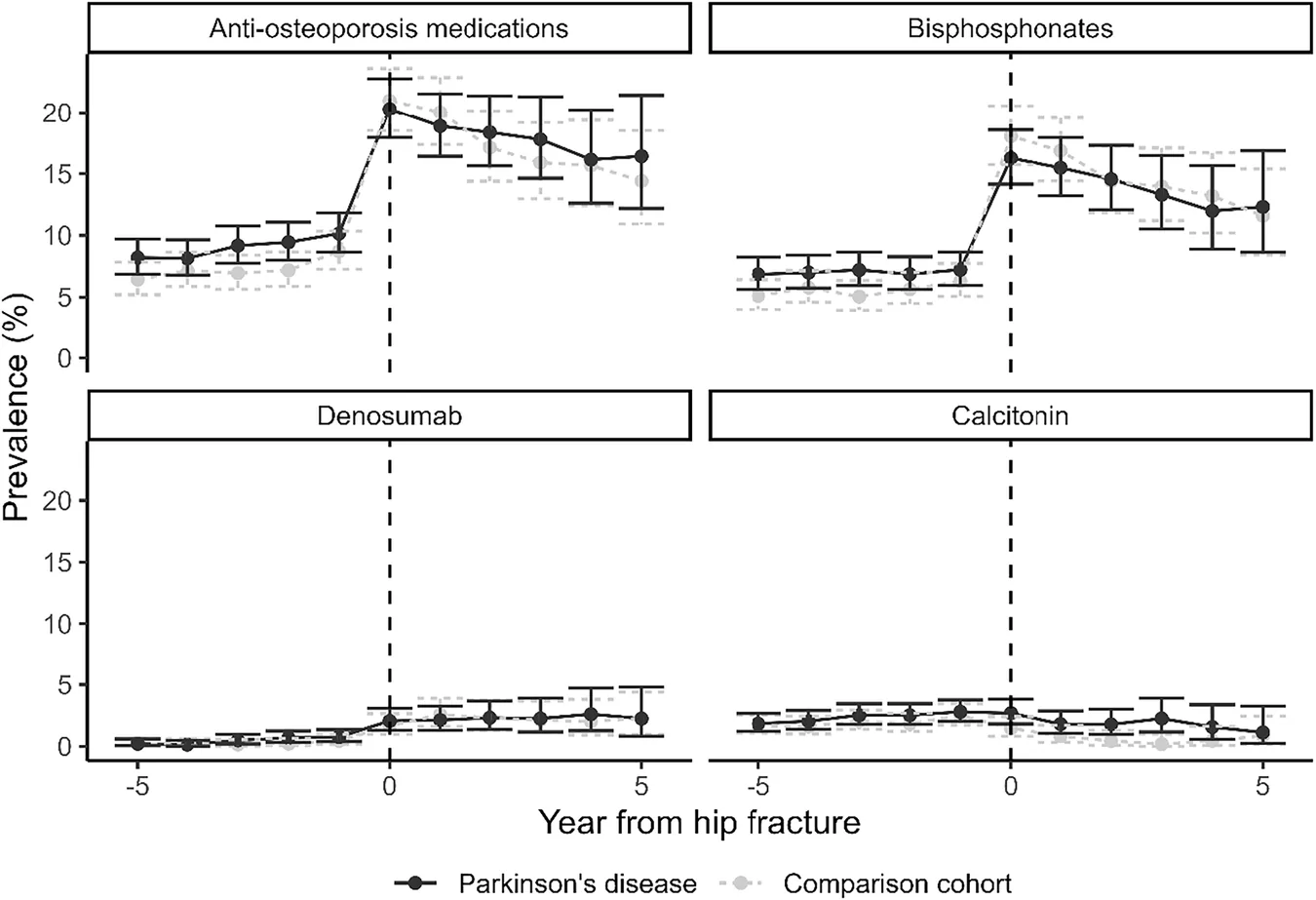
Annual prevalence of dispensing (from 5 years before to 5 years after hip fracture) in persons with and without Parkinson’s disease and had a hip fracture after diagnosis of Parkinson’s disease

Among our matched cohort, 1475 persons with PD and 1371 comparison persons had a hip fracture after index date. More females had a hip fracture than males in both persons with (58%, *n* = 855) and without PD (62%, *n* = 848) PD (Table S3). The median age at hip fracture was lower for persons with PD at 80 years (IQR 75–84) than persons without PD at 84 years (IQR 79–88) (Table S3). However, despite differences in demographics, persons with and without PD had similar prevalences for all anti-osteoporosis medications from 5 years before (PD: 8.20%, 95% CI 6.85–9.72%; without PD: 6.42%, 95% CI 5.18–7.85%) to 5 years after (PD: 16.4%, 95% CI 12.2–21.4%; without PD: 14.4%, 95% CI 10.9–18.5%) hip fracture (Fig. 2, Table S4). The prevalence of any anti-osteoporosis medications peaked within one year after hip fracture (PD: 20.3%, 95% CI 18.0–22.9%; without PD: 21.0% 95% CI 18.5–23.6%) (Fig. 2, Table S4). While females with PD had higher prevalence of anti-osteoporosis medications within one year after hip fracture than males with PD (females with PD: 25.6%, 95% CI 22.4–30.1%; males with PD: 12.2%, 95 CI% 8.31–14.7%), the post-fracture prevalence of anti-osteoporosis medications in males remained similarly elevated up to 5 years after hip fracture (12.3%, 95% CI 5.80–22.1%), instead of decreasing from hip fracture onwards till end of follow-up in females with PD (18.0%, 95% CI 12.8–24.1%) (Fig. S3, Table S4).

## Discussion

Our study found that the use of anti-osteoporosis medications was higher among persons with PD starting from 3 years before PD diagnosis, but the prevalence decreased after peaking at 4 years after diagnosis. Persons with and without PD had similar use of anti-osteoporosis medications around hip fractures, with 1 in 5 receiving a dispensing of an anti-osteoporosis medication within 1 year after hip fracture.

The heightened use of anti-osteoporosis medications from 3 years before PD diagnosis coincided with the higher risk of hip fracture we found in persons with PD from 3 years before diagnosis in our previous study [11]. The increased may be due to more persons with PD experiencing fractures and subsequently being screened and treated for osteoporosis [6]. Our current finding of increased use of anti-osteoporosis medications after hip fractures support this hypothesis. It may also be due to an increased fall risk observed in persons presenting with prodromal motor and nonmotor symptoms of PD [4, 16]. Reduced mobility and use of psychotropics, which we have previously found to be higher among persons with PD up to 7 years before PD diagnosis in this cohort, are important risk factors for hip fractures [17].

We had previously found that the elevated risk of hip fracture was sustained up to 10 years after PD diagnosis [11]. The decrease in prevalence of bisphosphonate use beyond 4 years after PD diagnosis may reflect the low adherence and persistence of osteoporosis pharmacotherapy [18, 19]. On the other hand, anti-osteoporosis medications should be targeted to older persons with previous fractures and low bone density, and a drug holiday of 1–2 years is recommended for persons with low risk of fractures, following 3–5 years of bisphosphonate treatment to minimize the risk of adverse events from long-term use [20]. Therefore, the decrease may also be due to clinicians’ decision on not prescribing or prescribing a drug holiday for bisphosphonate users, instead of individual users’ non-compliance. Future studies are needed to understand whether the decrease in usage was intended and adequate.

Pharmacological secondary prevention after hip fractures was low, peaking within 1 year following hip fracture at 20%. While the prevalence was comparable to the prevalence of other Nordic countries [21], it was much lower than the >50% prevalence reported in the United Kingdom [21, 22]. This may be due to differences in recommendations of national guidelines. Finnish Current Care Guideline on hip fracture recommends anti-osteoporosis medications only for patients who are likely to achieve independent mobility only, but not if patients are expected to be bedbound [17]. The guideline also emphasizes the need for fall risk management, highlighting that 90% of hip fractures resulted from a fall. Provision of multi-disciplinary fracture liaison services may improve secondary prevention, by managing falls risk, as well as raising the use of anti-osteoporosis medications following hip fractures [23, 24].

Highest prevalence of anti-osteoporosis medications peaked at 8 years after diagnosis of PD for males but 4 years after diagnosis of PD for females. This may be due to the earlier onset of osteoporosis in females due to postmenopausal bone loss, leading to an earlier use and subsequent discontinuation of anti-osteoporosis medications to reduce risk from long-term therapy [25].

The main strength of our study is the use of nationwide healthcare registers that minimize selection bias. Another strength of our study was the long follow-up of 15 years for each cohort member. The prevalence of anti-osteoporosis medications before the diagnosis of PD has not been studied previously. The prediagnostic stage was pertinent for persons with PD as our previous study found the risk of hip fracture was elevated before the diagnosis of PD [11]. Our study also have several limitations. As most of the vitamin D and calcium would be dispensed without prescription, their use could not be captured with our database. We also did not have information on blood test or scan results, e.g., bone mineral density, levels of vitamin D. Therefore, we could not evaluate whether adequate supplementation or treatment according to bone health was received. We did not have information on medication dispensed in hospitals, therefore we could not evaluate medications administered intravenously in hospitals or health care units, e.g., zoledronic acid.

## Conclusion

Our study found a higher prevalence of anti-osteoporosis medications among persons with PD from 3 years before diagnosis and similar anti-osteoporosis medication usage before and after their hip fractures among persons with and without PD in Finland. Our results suggest that other preventive measures, e.g., decreasing fall risk, in addition to anti-osteoporosis medications are needed to mitigate the doubled risk of hip fracture among persons with PD.

## Supplementary information

Supplementary table and figures
