# Supplementary material for: Anti-osteoporosis medications in persons with and without Parkinson disease in Finland
Source: Arch Osteoporos. 2026 Oct 5;21(1):143. doi: 10.1007/s11657-026-01780-z (PMC13638689; doi:10.1007/s11657-026-01780-z)
Supplement: Supplementary file 1 [file 11657_2026_1780_MOESM1_ESM.docx]

**Table S1.** Comorbidities diagnoses

|  | **ICD-10** | **ICD-9** | **Special reimbursement register code** | **Prescription register ATC code** |
| --- | --- | --- | --- | --- |
| Cardiovascular diseases (hypertension, coronary artery disease, chronic heart failure) | I10-I15, I20-I25, I42, I43, I50 | 401-405, 410, 411, 413, 4148, 4149, 4230A, 4298X, 4295A, 4296A, 4149C, 4121X, 4148X, 4140X, 4120X, 4428X, 4250A, 4251A, 4254A, 4255A, 4258A, 4259X, 4289X, 4273A | 205, 206, 213, 280, 201 |  |
| Diabetes |  |  | 103 | A10 (excluding A10BX01) |
| Stroke | I60-I64, I69 | 430-432 |  |  |
| Renal failure | I13.1, N18, N19, Z94.0, Z99.2, Z49 | 40311, 40391, 40412, 40492, 585, 586, V420, V451, V560, V568 | 137,138 |  |
| Rheumatoid arthritis |  |  | 202 |  |
| Asthma/ chronic obstructive pulmonary disease | J44-J46 | 4912A, 4960A, 4930A, 4931A, 4939A, 4939X, 4939B | 203 |  |
| Osteoporosis | M80, M81 | 7330 |  | M05BA, M05BB, M05BC, M05BX |
| Previous fractures (any hospital-treated fracture excluding femoral fracture) | S02, S12, S22, S32, S42, S52, S62, S82, S92, T02 |  |  |  |
| Substance abuse | F10-F19, G62.1, G31.2, G72.1, I42.6, K29.2, K70-K70.4, K70.9, K86.0, X45 Y15 T51.0 T51.1 T51.9, T40.0-T40.9, R78.0-R78.4 | 291, 292, 303, 304, 305, 3575A, 4255A, 5353A, 5710A, 5711A, 5712A, 5713X, 5770D, 5771C, 5771D, 9800A, 9801A, 9809X, 9650, 9696, 9697, 9698 |  |  |
| Note: ICD-9 codes were used to identified comorbidities from the Care register of Healthcare from 1987 until 1995 and ICD-10 codes from 1996 until 2016. The special reimbursement register data was available from 1972 and the prescription register data from 1995.  Abbreviations: ICD-10 and ICD-9, International Classification of Diseases 9th and 10th Revision; ATC, the Anatomical Therapeutic Chemical codes; CVD, cardiovascular diseases. | | | | |

**Table S2.** Baseline characteristics of persons with and without Parkinson’s disease at index date.

| **Characteristic** | **Parkinson's disease**  N = 11,051 | **Comparison cohort**  N = 22,102 | **p-value***^1^* | |
| --- | --- | --- | --- | --- |
| Sex, n (%) |  |  |  | |
| Female | 5,051 (46) | 10,102 (46) |  | |
| Male | 6,000 (54) | 12,000 (54) |  | |
| Age at index date, median (IQR) | 72 (64, 78) | 72 (64, 78) |  | |
| Age category at index date, n (%) |  |  |  | |
| <70 | 4,681 (42) | 9,397 (43) |  | |
| 70-79 | 4,574 (41) | 9,126 (41) |  | |
| 80+ | 1,796 (16) | 3,579 (16) |  | |
| Comorbidities, n (%) |  |  | | |
| Cardiovascular diseases | 4,958 (45) | 9,648 (44) | 0.036 | |
| Diabetes | 1,352 (12) | 2,739 (12) | 0.7 | |
| Stroke | 546 (4.9) | 799 (3.6) | <0.001 | |
| Renal failure | 56 (0.5) | 102 (0.5) | 0.6 | |
| Rheumatoid arthritis | 381 (3.4) | 872 (3.9) | 0.025 | |
| Asthma/ chronic obstructive pulmonary disease | 916 (8.3) | 2,000 (9.0) | 0.021 | |
| Osteoporosis | 686 (6.2) | 1,028 (4.7) | <0.001 | |
| Previous fractures | 595 (5.4) | 1,116 (5.0) | 0.2 | |
| Substance abuse | 144 (1.3) | 269 (1.2) | 0.5 | |
| *^1^*Generalized linear mixed model | | | |  |

Table S3. Baseline characteristics at index date of persons with and without PD who had an incident hip fracture.

| **Characteristic** | **Parkinson's disease**  N = 1,475 | **Comparison cohort**  N = 1,371 | **p-value***^1^* |
| --- | --- | --- | --- |
| Sex, n (%) |  |  | 0.035 |
| Female | 855 (58) | 848 (62) |  |
| Male | 620 (42) | 523 (38) |  |
| Age at hip fracture, median (IQR) | 80 (75, 84) | 84 (79, 88) | <0.001 |
| Age at index date, median (IQR) | 74 (68, 79) | 77 (71, 81) | <0.001 |
| Age category at index date, n (%) |  |  | <0.001 |
| <70 | 469 (32) | 262 (19) |  |
| 70-79 | 719 (49) | 721 (53) |  |
| 80+ | 287 (19) | 388 (28) |  |
| Time since index date (year), median (IQR) | 6.2 (3.5, 9.0) | 7.6 (3.9, 11.2) | <0.001 |
| Comorbidities, n (%) | | | |
| Cardiovascular diseases | 826 (56) | 885 (65) | <0.001 |
| Diabetes | 219 (15) | 284 (21) | <0.001 |
| Stroke | 123 (8.3) | 199 (15) | <0.001 |
| Renal failure | 20 (1.4) | 36 (2.6) | 0.015 |
| Rheumatoid arthritis | 64 (4.3) | 84 (6.1) | 0.032 |
| Asthma/ chronic obstructive pulmonary disease | 154 (10) | 192 (14) | 0.004 |
| Osteoporosis | 246 (17) | 166 (12) | <0.001 |
| Previous fractures | 274 (19) | 259 (19) | 0.8 |
| Substance abuse | 13 (0.9) | 26 (1.9) | 0.020 |
| *^1^*Linear mixed model for continuous variables; generalized linear mixed model for categorical variables | | | |

**Table S4.** Prevalence of anti-osteoporosis medications per year of follow-up from hip fracture.

| **Cohort** | **Year from hip fracture** | **Overall** | | | **Males** | | | **Females** | | |
| --- | --- | --- | --- | --- | --- | --- | --- | --- | --- | --- |
|  |  | **Population** | **User** | **Prevalence  (%, 95% CI)** | **Population** | **User** | **Prevalence  (%, 95% CI)** | **Population** | **User** | **Prevalence  (%, 95% CI)** |
| Comparison cohort | -5 | 1370 | 88 | 6.42 [5.18 - 7.85] | 523 | 9 | 1.72 [0.79 - 3.24] | 847 | 79 | 9.33 [ 7.45 - 11.49] |
| Parkinson's disease | -5 | 1475 | 121 | 8.20 [6.85 - 9.72] | 620 | 6 | 0.97 [0.36 - 2.09] | 855 | 115 | 13.45 [11.23 - 15.92] |
| Comparison cohort | -4 | 1369 | 98 | 7.16 [5.85 - 8.65] | 522 | 9 | 1.72 [0.79 - 3.25] | 847 | 89 | 10.51 [ 8.52 - 12.77] |
| Parkinson's disease | -4 | 1474 | 120 | 8.14 [6.80 - 9.66] | 619 | 12 | 1.94 [1.01 - 3.36] | 855 | 108 | 12.63 [10.48 - 15.05] |
| Comparison cohort | -3 | 1369 | 95 | 6.94 [5.65 - 8.42] | 523 | 14 | 2.68 [1.47 - 4.45] | 846 | 81 | 9.57 [ 7.68 - 11.76] |
| Parkinson's disease | -3 | 1472 | 135 | 9.17 [7.75 - 10.76] | 618 | 16 | 2.59 [1.49 - 4.17] | 854 | 119 | 13.93 [11.68 - 16.44] |
| Comparison cohort | -2 | 1366 | 98 | 7.17 [5.86 - 8.67] | 521 | 9 | 1.73 [0.79 - 3.25] | 845 | 89 | 10.53 [ 8.54 - 12.80] |
| Parkinson's disease | -2 | 1471 | 139 | 9.45 [8.00 - 11.06] | 618 | 22 | 3.56 [2.24 - 5.34] | 853 | 117 | 13.72 [11.48 - 16.21] |
| Comparison cohort | -1 | 1366 | 119 | 8.71 [7.27 - 10.33] | 520 | 17 | 3.27 [1.92 - 5.18] | 846 | 102 | 12.06 [ 9.94 - 14.44] |
| Parkinson's disease | -1 | 1468 | 149 | 10.15 [8.65 - 11.81] | 616 | 25 | 4.06 [2.64 - 5.93] | 852 | 124 | 14.55 [12.25 - 17.10] |
| Comparison cohort | 0 | 1025 | 215 | 20.98 [18.52 - 23.60] | 359 | 38 | 10.58 [7.60 - 14.24] | 666 | 177 | 26.58 [23.26 - 30.11] |
| Parkinson's disease | 0 | 1119 | 227 | 20.29 [17.97 - 22.76] | 411 | 46 | 11.19 [8.31 - 14.65] | 708 | 181 | 25.56 [22.39 - 28.95] |
| Comparison cohort | 1 | 854 | 171 | 20.02 [17.39 - 22.87] | 284 | 26 | 9.15 [6.07 - 13.13] | 570 | 145 | 25.44 [21.91 - 29.22] |
| Parkinson's disease | 1 | 943 | 178 | 18.88 [16.43 - 21.52] | 331 | 35 | 10.57 [7.48 - 14.40] | 612 | 143 | 23.37 [20.07 - 26.92] |
| Comparison cohort | 2 | 695 | 119 | 17.12 [14.39 - 20.13] | 222 | 20 | 9.01 [5.59 - 13.57] | 473 | 99 | 20.93 [17.35 - 24.88] |
| Parkinson's disease | 2 | 735 | 135 | 18.37 [15.63 - 21.36] | 233 | 26 | 11.16 [7.42 - 15.92] | 502 | 109 | 21.71 [18.18 - 25.58] |
| Comparison cohort | 3 | 566 | 90 | 15.90 [12.98 - 19.18] | 166 | 14 | 8.43 [4.69 - 13.75] | 400 | 76 | 19.00 [15.27 - 23.19] |
| Parkinson's disease | 3 | 534 | 95 | 17.79 [14.64 - 21.30] | 160 | 19 | 11.88 [7.30 - 17.92] | 374 | 76 | 20.32 [16.36 - 24.76] |
| Comparison cohort | 4 | 447 | 70 | 15.66 [12.42 - 19.37] | 130 | 11 | 8.46 [4.30 - 14.64] | 317 | 59 | 18.61 [14.48 - 23.34] |
| Parkinson's disease | 4 | 384 | 62 | 16.15 [12.61 - 20.21] | 103 | 10 | 9.71 [4.75 - 17.13] | 281 | 52 | 18.51 [14.14 - 23.55] |
| Comparison cohort | 5 | 354 | 51 | 14.41 [10.92 - 18.50] | 111 | 12 | 10.81 [5.71 - 18.12] | 243 | 39 | 16.05 [11.67 - 21.28] |
| Parkinson's disease | 5 | 268 | 44 | 16.42 [12.19 - 21.41] | 73 | 9 | 12.33 [5.80 - 22.12] | 195 | 35 | 17.95 [12.83 - 24.07] |

**Figure S1.** Study flow diagram

**Figure S2.** Annual prevalence of dispensing in males and females with and without Parkinson’s disease from 5 years before to 10 years after diagnosis of Parkinson’s disease.

**Figure S3.** Annual prevalence of dispensing in persons with and without Parkinson’s disease from 5 years before to 10 years after diagnosis of Parkinson’s disease, with additional censoring upon incident hip fracture.

**Figure S4.** Annual prevalence of dispensing (from 5 years before to 5 years after hip fracture) in males and females with and without Parkinson’s disease and had a hip fracture after diagnosis of Parkinson’s disease.
